# The Sterilisation of Rubber Gloves

**Published:** 1900-12-01

**Authors:** 


### THE STERILISATION OF RUBBER GLOVES.

Dr. E. L. Kellogg[1] describes a method of sterilising rubber gloves which seems practical. He says that when he was an interne at Bellevue Hospital rubber gloves were used extensively and were sterilised in the following simple and effective manner:—The gloves were filled with gauze, each finger being carefully packed so that two layers of rubber were nowhere in contact. They were wrapped separately in several layers of gauze, and were then boiled in plain water for 20 minutes. From this they were transferred to cold perchloride solution (1 to 2,000), where they were left until ready for use. The difficulty experienced in putting on wet gloves was easily overcome by filling them with solution and allowing the hand to force it out as the gloves were drawn on.

[1] New York Medical Record.

